# Effect of Race and Ethnicity on Academic Achievements in Cancer Physicians and Scientists

**DOI:** 10.3390/curroncol33060321

**Published:** 2026-05-29

**Authors:** Doreen A. Ezeife, Amanda Khan, Mark Melika-Abusefien, Edouarda Taguedong, Md Mahsin, Shaun K. Loewen

**Affiliations:** 1 Department of Oncology, Arthur J. E. Child Comprehensive Cancer Centre, University of Calgary, Calgary, AB T2N 5G2, Canada; amanda.khan@ahs.ca (A.K.); md.mahsin@albertahealthservices.ca (M.M.); shaun.loewen@albertahealthservices.ca (S.K.L.); 2 School of Medicine, University of Galway, H91 V4AY Galway, Ireland; markmelika03@gmail.com; 3 School of Medicine, McGill University, Montreal, QC H3A 0G4, Canada; edouarda.taguedong@mail.mcgill.ca

**Keywords:** equity, oncology, race, ethnicity, academic rank

## Abstract

**Background:** Diversity in academia promotes research that can reduces health disparities and addresses equity issues for marginalized populations. This study aims to examine the effect of visible minority status on academic achievements in cancer physicians and scientists. **Methods:** Faculty at the tertiary cancer center in Calgary, Alberta, Canada, completed a survey in 2023 to evaluate demographics, academic rank, leadership positions, number of trainees mentored, number of publications, and amount of grant funding. Chi-square tests and regression analyses examined the impact of race and ethnicity on these academic achievements. **Results:** The survey was completed by 74 faculty members (47% male, 43% female, 9% gender fluid or providing no answer) with a response rate of 26%. Seven percent were Black or Latin American, 18% East Asian or Southeast Asian, 19% West or South Asian, 39% Caucasian, 6% mixed race, and 11% not providing an answer. Visible minorities were underrepresented in the full professor rank (19%) compared to non-visible minorities (38%) and were overrepresented in assistant/associate professors (28% and 53%, respectively), with 41% of non-visible minorities having the title of assistant professor and 21% as associate professor (*p* = 0.02). Visible minorities were less likely to have both parents college-educated (OR 0.30, 95% CI 0.09–0.92, *p* = 0.042) and also less likely to have been raised in a home with household income above $100,000 (OR 0.26, 95% CI 0.07–0.90, *p* = 0.040). **Discussion:** Visible minorities are underrepresented in the full professor academic rank. Larger studies are needed to evaluate whether race and ethnicity significantly impact achievements in oncology academics.

## Simple Summary

Having a diverse group of people working in academic settings helps lead to research that better understands and reduces health gaps for underserved or disadvantaged communities. However, prior studies suggest that people from racial and ethnic minority groups may face barriers to career advancement in academic medicine. This study looked at whether race or ethnicity affects career success among cancer doctors and researchers in Calgary. In 2023, 74 faculty members completed a survey about their background, academic rank, research output, and leadership roles. The results showed that academics from visible minority groups were less likely to be full professors and were more often in junior or mid-level roles. They were also less likely to come from higher-income or highly educated family backgrounds. Overall, the findings suggest ongoing disparities in academic advancement, and larger studies are needed to better understand these differences.

## 1. Background

Diversity in academic medicine results in high-quality research and improvement of physician cultural competence [1]. While diversity in medical faculty has improved over the years, some visible minorities remain underrepresented in medicine and experience inequivalent academic success compared to their Caucasian counterparts. The proportion of women and minorities among academic faculty members may not be reflected in the proportion of staff getting promoted and taking on leadership positions. Prior studies have shown that physicians who identify as Black, Hispanic, or Native American have lower rates of promotion compared to their Asian and White peers [2].

Oncology has been shown to be one of the least diverse subspecialties in medicine [3]. Additionally, racial and ethnic minority patients experience higher cancer incidence and mortality largely due to access issues, distrust in the medical system based on historical factors, and lower rates of patient enrollment in clinical trials [4,5]. Improving diversity in the physician workforce is a system intervention that can address these equity gaps, although ensuring that proportional increases are seen in academic achievements is also equally important.

Achievements such as academic promotion, leadership roles, scholarly productivity with journal publications, grant funding, and providing mentorship improve decision-making freedom and job security and have been associated with higher career satisfaction [6]. Obtaining academic achievements, such as promotion, have been correlated with increased job control, which can ensure physician workforce stability in the long term. Physicians who feel more satisfied and in control of their jobs are less likely to leave academic medicine. In some institutions, these academic advancements can also be tied to increased compensation. Lack of mentorship and role models has also been identified as a contributor to gender equity issues in academic medical subspecialties, and this issue affects female physicians more than males [7]. The Canadian 2021 census defined visible minorities as being a member of one of the following groups: Arab, Black, Caucasian, Chinese, Filipino, Indigenous, Japanese, Korean, Latin American, South Asian, Southeast Asian, or West Asian. The purpose of this study was to examine the impact of visible minority status on the achievement of academic milestones among oncologists and scientists at a large tertiary cancer center. This study was conducted in Calgary, Alberta, where the population is approximately 1.5 million people, with about 40% being visible minorities [8]. Academic physicians and scientists are recruited to Calgary from other centers across Canada or other countries, and once hired, they have an appointment at the University of Calgary. They can apply for academic promotion when they have demonstrated performance in research with publications and/or grants, teaching, and service on administrative committees, editorial boards, or academic leadership. Visible minority status is not factored into academic promotion decisions.

## 2. Methods

Survey participants were eligible if they were physicians and/or scientists at the Tom Baker Cancer Center (TBCC, now Arthur J.E. Child Comprehensive Cancer Center) in Calgary, Alberta, Canada. Collected participant demographic information included age, marital status, number of children and other dependents, childhood household income, academic rank, stage in career, degrees earned, sexual orientation, identifying as having a disability, visible minority status, and religion. Participants were classified as a visible minority if they responded “definitely yes” or “probably yes” to the following question: “From your name, physical appearance, and/or anything you always wear, are you easily identifiable as a visible minority?” Race/ethnicity categories for visible minorities were defined using the 2016 Canadian census population definitions. Participants could choose as many identifiers as they felt represented them. Participants were classified as having a leadership position if they self-reported holding any leadership position at the departmental, institute, university, national, or international levels. Data on grant funding, number of mentees, leadership roles, and publications were also self-reported. The online survey instrument can be found in Supplementary File S1 and was hosted via Qualtrics (https://www.qualtrics.com/ (accessed on 12 December 2023). The survey comprised 39 multiple-choice questions and 4 short-answer questions. The survey took 10–15 min to complete, so participants were expected to complete it in one sitting. All physicians and scientists at the TBCC were invited by email to complete the survey. An initial email was sent out to complete the survey, and two reminder emails were sent out two and four weeks later. The study received ethics approval from the Health Ethics Research Board of Alberta; study Id: HREBA.CC-22-0145_REN1. The survey was anonymous and voluntary, and all participants provided consent by choosing to complete the survey. Participants were recruited from September to November 2023.

Statistical analyses were conducted on the survey results. Descriptive statistics and frequency tabulation were used to summarize the demographic variables. Chi-square tests were used to examine associations between visible minority status and academic rank or leadership roles. Multivariable logistic regression analyses were conducted to determine whether there was an association between visible minority status (independent variable) and the following dependent variables: educational level of parents, household income, degrees earned, number of trainees mentored, number of peer-reviewed publications, and grant funding. Linear regression analyses studied the association between visible minority status and the number of trainees mentored while controlling for academic rank, age group, and gender. The statistical significance level was set at 0.05 or lower for all statistical analyses. All statistical analyses were conducted (M.M.) using *R* software (version 4.4.1; R Core Team, 2024 [9]).

## 3. Results

Table 1 shows the demographic baseline characteristics. A total of 74 oncology faculty completed the survey, which corresponded to a response rate of 26% of the total cohort of 282 cancer physicians and scientists who were invited to participate. The sex distribution was 35 males (52%), 32 females (48%), and 7 individuals who identified as either gender fluid or preferred not to answer. Sixteen (21%) of the survey respondents were physicians, while the remainder were PhD scientists. Survey respondents were mostly equally distributed across academic rank, with 25 respondents (36%) ranked as assistant professors, 25 respondents (36%) were associate professors, and 19 respondents (28%) were full professors. Thirty-two (48%) survey respondents identified as a visible minority, and 29 (44%) of survey respondents identified as Caucasian.

### 3.1. Visible Minority Status, Academic Rank, and Leadership Roles

**Figure 1:**
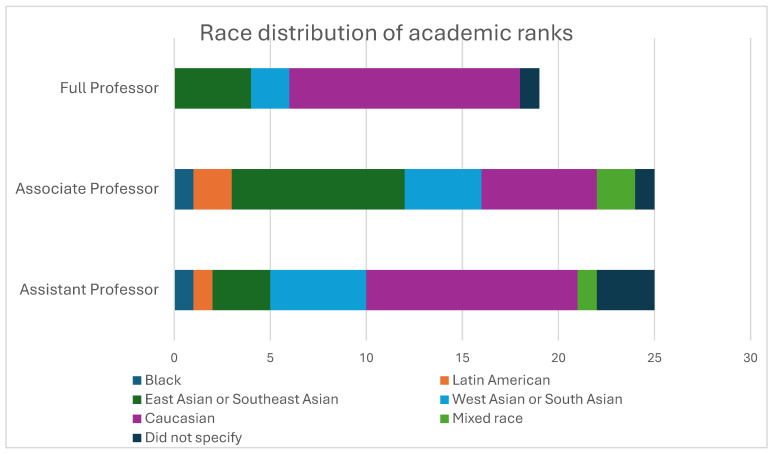
Race distribution by academic rank.

Figure 1 shows the race/ethnicity distribution of the survey respondents by academic rank. There was a statistically significant disparity in representation of visible minorities at the full professor rank, with visible minorities comprising only 19% of full professors. Visible minorities were overrepresented in associate professors (53%) and modestly represented in assistant professors (28%) (*p* = 0.02, Table 2). The non-visible minorities were overrepresented as full professors (38%), with 21% as associate professors and 41% as assistant professors. There was no association between proportions of visible minorities and non-visible minorities that were older or senior in their career (*p* = 0.83, *p* = 0.97, respectively; Table 2). There was no significant association between leadership roles and visible minority status (*p* = 0.92, Table 2).

### 3.2. Visible Minority Status and Childhood Factors

Visible minorities were less likely to have both parents with a college or university degree compared to their non-visible minority counterparts, while keeping factors like academic rank, age group, and gender constant (OR 0.30, 95% CI 0.09–0.92, *p* = 0.042, Table 3). Visible minorities were also less likely to have been raised in a home with a household income >$100,000 (OR 0.26, 95% CI 0.07–0.90, *p* = 0.040, Table 4).

### 3.3. Visible Minority Status and Academic Achievements

There was no significant association between visible minority status and earning a PhD degree (OR 0.52, 95% CI 0.11–2.24, *p* = 0.4). Linear regression analyses showed that there was a trend towards visible minorities training 1.8 times fewer mentors (Beta −1.8, 95% CI −3.7–0.21, *p* = 0.085) when controlling for academic rank, age group, and gender. There was a trend towards visible minorities being less likely to have obtained grant funding >$100,000 in the past 5 years (OR 0.35, 95% CI 0.09–1.15, *p* = 0.092). There was no association between visible minority status and number of peer-reviewed publications authored (OR 0.40, 95% CI 0.08–1.65, *p* = 0.2).

## 4. Discussion

Research investigating the impact of race and ethnicity on academic achievements in oncology is limited. Our study is the first to examine the impact of race on academic career advancements in oncology faculty. Our results indicate that the proportion of non-visible minorities who are full professors is double that of the visible minorities in our survey participants. Older and more senior-career academics are more likely to be full professors, yet our study did not show lower proportions of visible minorities in older or senior-career participants. Thus, age and career seniority cannot explain the lower proportions of visible minorities who were full professors. Racial inequity in academic career milestones has been previously reported in other medical specialties, which have shown that Caucasian faculty and men are more likely to be full professors compared to women and visible minorities [10]. Our results also showed a trend towards oncology faculty who were visible minorities mentoring fewer trainees and having less grant funding. In gastroenterology, an analysis of professor rank by sex and race found increasing underrepresentation of women, Blacks, and Hispanics, as academic rank advanced from assistant to full professor [11]. Lack of retention was hypothesized as a potential reason for this inequity, as supported by the low ratios of associate to assistant professors and full to associate professors. Promotion of faculty of color has been shown to occur at lower rates, and there is greater faculty attrition from academic medicine in comparison to Caucasian faculty [12].

There are other potential reasons for visible minorities being less likely to have the highest rank in academic medicine, the full professor title. Minorities may seek academic promotion less frequently than their Caucasian counterparts due to imposter syndrome, lack of mentorship from senior colleagues, recent migration to Canada, or lack of protected academic time. It is also possible that those minorities who do apply for promotion may be less likely to succeed due to institutional discrimination. There is extensive prior work demonstrating that minorities in academia experience different success rates in their academic endeavors after adjusting for productivity and other key factors. In a study examining National Institutes of Health (NIH) grant funding success rates by race, Asian and Black women PhDs and MDs were less likely to receive funding, partly because the submission rate was lower for women [13]. Abdalla et al. also showed that over the past 30 years, there was an increase in the number of Black physicians in the workforce, but this increase did not coincide with an increase in Black and Hispanic physician authorship in medical journals across all clinical specialties [14]. Institutional biases may result in more visible minorities and women being hired into positions with high service demands that leave little protected time for research, mentoring, and grant applications. High service positions can include non-tenure-track academic jobs for scientists or job profiles with heavier clinical loads for physicians. The persistence of institutional biases is exemplified in several areas, from speaker introduction data showing that women are less likely than men to be introduced by their titles to leadership positions in many academic centers being mostly held by males and Caucasians [15]. To combat institutional biases, focused efforts can reduce the impact of implicit bias in recruitment and delegation of leadership roles, and mentorship of early-career faculty members can close equity gaps and create a diverse and productive workforce [16].

Our study did not find any significant association between visible minority status and leadership roles or publications authored. We showed that there was a trend for visible minorities to mentor fewer students and receive less grant funding than non-visible minorities. Several studies have previously shown that women and minorities are underrepresented in research authorship, conference speakers, and journal editorial boards [17,18,19]. In a study by Abdalla et al., under 30% of journal articles in the New England Journal of Medicine (NEJM) had a female first author [20]. Black senior authorship in NEJM was seen in only 6.9% of articles in 2019, and Hispanic first authorship was 3.9% in 2016, with little change over the past three decades. Racial disparities in the achievement of academic milestones have been reported for several specialties, including ophthalmology, internal medicine, neurosurgery, hematology, neurology, emergency medicine, and plastic surgery, among others [18,19,21,22,23,24,25]. Individual academic achievements are not independent of one another, so visible minorities who are not publishing or receiving grant funding are less likely to apply for promotion. Thus, discrimination and racial disparities in one realm can have a ripple effect to impact other areas of an academic’s career, and the effects can be lasting and pronounced. Mentorship can provide junior faculty with professional advice and tools to help them get grant funding, acquire leadership positions, and publish research that can increase academic productivity and lead to promotion. Strong and rigorous mentorship of early faculty is particularly helpful for visible minorities who may not have academic faculty members already exemplified in their social networks. Prior data showed that having a mentor significantly increased the likelihood of promotion to professor in academic faculty, which further underscores the importance of career mentorship of early- and mid-career faculty in order to bridge equity gaps [26].

### Limitations

Interpreting the findings of our study should be done in the context of our study’s main limitation, which was the survey design, low survey-response rates, and small sample size. Our findings of a trend towards fewer mentees and less grant funding for visible minorities suggest that a larger study may provide more insight into how race and ethnicity impact these measures of academic productivity. Our small sample size also did not allow for an analysis of the intersectionality of race and gender on academic achievements. The self-reported nature of our data and responder bias can increase susceptibility for over- or underestimation by respondents, although our main finding of racial underrepresentation in academic promotion likelihood is consistent with prior work. The survey-response rate was 26%, which is similar to prior survey studies conducted post-COVID-19 pandemic, where survey fatigue can be prevalent [18].

## 5. Conclusions

Our research shows that visible minorities are underrepresented in full professorships in academic oncology. Racial imbalances persist in the achievement of this important career milestone. Our study findings on the effect of race on academic career advancement warrant ongoing investigation along with the intersectionality of race and gender in academic achievements. Future work can determine the causal forces driving these imbalances and can help shape the design of programs that foster mentorship for young minority physicians and scientists in academia.

## Figures and Tables

**Table 1:** Baseline characteristics of cancer physician and researcher demographics.

	Overall *n* (%)
	*N* = 74
Academic Rank	
Assistant Professor	25 (36%)
Associate Professor	25 (36%)
Full professor	19 (28%)
Did not specify	5
Age group	
25–44	30 (43%)
45–54	28 (41%)
≥55	11 (16%)
Did not specify	5
Marital status	
Married	62 (90%)
Non-married	7 (10%)
Did not specify	5
Childhood household income	
<$100,000	39 (60%)
>$100,000	26 (40%)
Did not specify	9
Degrees earned	
Non-PhD	56 (81%)
PhD	13 (19%)
Did not specify	5
Parents college-educated	
Both	36 (55%)
Not both	30 (45%)
Did not specify	8
Grant funding obtained	
≤$100,000	24 (36%)
>$100,000	42 (64%)
Did not specify	8
Publications authored	
<25	25 (38%)
≥25	41 (62%)
Did not specify	8
Gender	
Man	35 (52%)
Woman	32 (48%)
Gender fluid	1
Did not specify	6
Visible minority	
Yes	32 (48%)
No	34 (52%)
Did not specify	8
Race	
Caucasian	29 (44%)
Non-Caucasian Black or Latin American East Asian or Southeast Asian West Asian or South Asian Other (“mixed” or “I prefer not to answer”) Did not specify	37 (56%) 5 16 10 6 8

**Table 2:** Representation of visible minorities in different academic ranks.

	Visible Minority (No) *N* = 34 (%)	Visible Minority (Yes) *N* = 32 (%)	Chi-Squared *p*-Value
**Academic rank**			
Assistant Professor	14 (41%)	9 (28%)	0.020
Associate Professor	7 (21%)	17 (53%)	
Full professor	13 (38%)	6 (19%)	
**Age**			
≥ 55	5 (14.7%)	6 (18.8%)	0.83
45–54	15 (44.1%)	12 (37.5%)	
25–44	14 (41.2%)	14 (43.8%)	
**Career stage**			
Early career (<5 years)	6 (17.6%)	5 (15.6%)	0.97
Mid-career (5–15 years)	14 (41.2%)	14 (43.8%)	
Senior career (>15 years)	14 (41.2%)	13 (40.6%)	
**Leadership roles**			
Any leadership title	23 (68%)	23 (72%)	0.92
No leadership title	11 (32%)	9 (28%)	

**Table 3:** Multivariable analysis examining the association between visible minority status and both parents having a college/university degree, while adjusting for academic rank, age, and gender. OR = Odds Ratio, CI = Confidence Interval.

	OR	95% CI	*p*-Value
**Visible minority status**			
No	—	—	
Yes	0.30	0.09, 0.92	0.042
**Academic rank**			
Assistant Professor	—	—	
Associate Professor	2.07	0.55, 8.63	0.3
Full professor	1.03	0.20, 4.95	>0.9
**Age**			
≥ 55	—	—	
25–44	0.26	0.04, 1.56	0.2
45–54	0.29	0.05, 1.49	0.2
**Gender**			
Man	—	—	
Woman	1.04	0.35, 3.08	>0.9

**Table 4:** Multivariable analysis examining the association between visible minority status and childhood household income above $100,000, while adjusting for academic rank, age, and gender. OR = Odds Ratio, CI = Confidence Interval.

	OR	95% CI	*p*-Value
**Visible minority status**			
No	—	—	
Yes	0.26	0.07, 0.90	0.040
**Academic rank**			
Assistant Professor	—	—	
Associate Professor	0.46	0.10, 2.03	0.3
Full professor	0.26	0.04, 1.51	0.15
**Age**			
≥ 55	—	—	
25–44	0.66	0.08, 5.46	0.7
45–54	0.32	0.04, 2.37	0.3
**Gender**			
Man	—	—	
Woman	5.17	1.46, 22.1	0.016

## Data Availability

The original contributions presented in this study are included in the article. Further inquiries can be directed to the corresponding author.

## Supplementary Materials

The following supporting information can be downloaded at: https://www.mdpi.com/article/10.3390/curroncol33060321/s1, File S1: The EROMA Survey study.

## References

[1] Brach C., Fraser I. (2002). Reducing disparities through culturally competent health care: An analysis of the business case. Qual. Manag. Healthc..

[2] Xierali I.M., Nivet M.A., Syed Z.A., Shakil A., Schneider F.D. (2021). Recent Trends in Faculty Promotion in U.S. Medical Schools: Implications for Recruitment, Retention, and Diversity and Inclusion. Acad. Med..

[3] Deville C., Chapman C.H., Burgos R., Hwang W.T., Both S., Thomas C.R. (2014). Diversity by Race, Hispanic Ethnicity, and Sex of the United States Medical Oncology Physician Workforce Over the Past Quarter Century. J. Oncol. Pract..

[4] Ezeife D.A., Padmore G., Vaska M., Truong T.H. (2022). Ensuring equitable access to cancer care for Black patients in Canada. CMAJ Can. Med. Assoc. J..

[5] Deville C., Charles-Obi K., Santos P.M.G., Mattes M.D., Hussaini S.M.Q. (2023). Oncology Physician Workforce Diversity: Rationale, Trends, Barriers, and Solutions. Cancer J..

[6] Johnston D.W., Lee W.S. (2013). Extra Status and Extra Stress: Are Promotions Good for Us?. ILR Rev..

[7] Mascarenhas A., Moore J.E., Tricco A.C., Hamid J., Daly C., Bain J., Jassemi S., Kiran T., Baxter N., Straus S.E. (2017). Perceptions and experiences of a gender gap at a Canadian research institute and potential strategies to mitigate this gap: A sequential mixed-methods study. CMAJ Open.

[8] Statistics Canada (2021). Census Profile, 2021 Census of Population: Calgary, Alberta. https://www12.statcan.gc.ca/census-recensement/2021/dp-pd/prof/details/page.cfm?Lang=E&SearchText=calgary&DGUIDlist=2021A00054806016,2021S051248061345,2021S051248060199&GENDERlist=1&STATISTIClist=1&HEADERlist=0.

[9] R Core Team (2024). R: A Language and Environment for Statistical Computing.

[10] Clark L., Shergina E., Machado N., Scheuermann T.S., Sultana N., Polineni D., Shih G.H., Simari R.D., Wick J.A., Richter K.P. (2024). Race and Ethnicity, Gender, and Promotion of Physicians in Academic Medicine. JAMA Netw. Open.

[11] Merchant J.L., Omary M.B. (2010). Underrepresentation of underrepresented minorities in academic medicine: The need to enhance the pipeline and the pipe. Gastroenterology.

[12] Satiani B., Williams T.E., Brod H., Way D.P., Ellison E.C. (2013). A review of trends in attrition rates for surgical faculty: A case for a sustainable retention strategy to cope with demographic and economic realities. J. Am. Coll. Surg..

[13] Ginther D., Kahn S., Schaffer W.T. (2016). Gender, Race/Ethnicity, and National Institutes of Health R01 Research Awards: Is There Evidence of a Double Bind for Women of Color?. Acad. Med..

[14] Abdalla M., Abdalla S., Maurer L.R., Ortega G., Abdalla M. (2024). American Black Authorship Has Decreased Across All Clinical Specialties Despite an Increasing Number of Black Physicians Between 1990 and 2020 in the USA. J. Racial. Ethn. Health Disparities.

[15] Duma N., Durani U., Woods C.B., Kankeu Fonkoua L.A., Cook J.M., Wee C., Fuentes H.E., Gonzalez-Velez M., Murphy M.C., Jain S. (2019). Evaluating Unconscious Bias: Speaker Introductions at an International Oncology Conference. J. Clin. Oncol..

[16] Newman E.A., Waljee J., Dimick J.B., Mulholland M.W. (2019). Eliminating Institutional Barriers to Career Advancement for Diverse Faculty in Academic Surgery. Ann. Surg..

[17] Ruzycki S.M., Fletcher S., Earp M., Bharwani A., Lithgow K.C. (2019). Trends in the Proportion of Female Speakers at Medical Conferences in the United States and in Canada, 2007 to 2017. JAMA Netw. Open.

[18] Lithgow K.C., Earp M., Bharwani A., Fletcher S., Ruzycki S.M. (2020). Association Between the Proportion of Women on a Conference Planning Committee and the Proportion of Women Speakers at Medical Conferences. JAMA Netw. Open.

[19] Patel S.R., Riano I., Abuali I., Ai A., Geiger G., Pimienta J., Roggio A.R., Dhawan N., Dizman N., Salinas A.L. (2023). Race/Ethnicity and Gender Representation in Hematology and Oncology Editorial Boards: What is the State of Diversity?. Oncologist.

[20] Abdalla M., Abdalla M., Abdalla S., Saad M., Jones D.S., Podolsky S.H. (2023). The Under-representation and Stagnation of Female, Black, and Hispanic Authorship in the Journal of the American Medical Association and the New England Journal of Medicine. J. Racial. Ethn. Health Disparities.

[21] Sirivolu S., Pike S., Reid M.W., Berry J.L., Chang M.Y., Nguyen A.M. (2025). Discrimination Within the US Ophthalmology Workforce. JAMA Ophthalmol..

[22] Sheppard G., McIlveen-Brown E., Jacques Q., Barry N., Morris J., Yi Y., Bischoff T., Pham C., Menchetti I., Lim R. (2024). Perceptions of gender equity in emergency medicine in Canada. Can. J. Emerg. Med..

[23] Patel S.I., Grewal P., Nobleza C.O.S., Ayub N., Ky K.-E., Kung D.H., Shah S., Abdennadher M., Alexander H.B., Frost N. (2024). Analysis of Faculty Gender and Race in Scholarly Achievements in Academic Neurology. J. Womens Health.

[24] Michel M., Peart R., Yan S.C., Still M.E.H., Melnick K., San A., Gonzalez B., Hodges T.R., Newman W.C., Mbabuike N. (2024). Academic accomplishments of Black neurosurgeons in the United States. J. Neurosurg..

[25] Ha G., Benyamein P., Reghunathan M., Vatsia S., Blum J., Gosman A.A. (2023). Racial and Ethnic Disparities in Selected Speakers at Plastic Surgery Conferences. Plast. Reconstr. Surg. Glob. Open.

[26] Wise M.R., Shapiro H., Bodley J., Pittini R., McKay D., Willan A., Hannah M.E. (2004). Factors affecting academic promotion in obstetrics and gynaecology in Canada. J. Obstet. Gynaecol. Can..

