# Supplementary material for: Effect of Race and Ethnicity on Academic Achievements in Cancer Physicians and Scientists
Source: Curr Oncol. 2026 May 29;33(6):321. doi: 10.3390/curroncol33060321 (PMC13297884; doi:10.3390/curroncol33060321)
Supplement: Supplementary file 1 [file curroncol-33-00321-s001.zip › curroncol-4207827-supplementary.pdf]

The EROMA Survey study

Start of Block: Default Question Block

Q1 **Impact of Race and Ethnicity on Academic Oncology Career Milestones Within the Department of Oncology and the Arnie Charbonneau Cancer Institute at the University of Calgary**

Researcher: Dr. Doreen Ezeife
Medical Oncologist, Tom Baker Cancer Centre
Clinical Assistant Professor, University of Calgary
Department of Oncology Equity, Diversity and Inclusion Lead
Lung Provincial Tumour Group Lead
Doreen.ezeife@albertahealthservices.ca
403-521-3093

Medical Resident Co-Investigator: Dr. Amanda Khan
PGY3 Radiation Oncology
University of Calgary
amanda.khan@albertahealthservices.ca
403-521-3642

Funder(s)/Sponsor: Arnie Charbonneau EDI Grant

WHY AM I BEING ASKED TO TAKE PART IN THIS RESEARCH STUDY?
You are being invited to participate in an anonymous research study because you are a current physician or scientist within the Department of Oncology or the Arnie Charbonneau Cancer Institute at the University of Calgary. The purpose of this study is to help understand the demographics of our oncology workforce in terms of race/ethnicity, gender identity, prevalence of disability and accommodation, sexual orientation and socioeconomic background and employee perceptions of discrimination and thoughts on workplace culture and environment.

Providing your email address at the end is optional, will not be linked to your individual survey responses, and is only needed to enter your name into a raffle for a chance to win Apple AirPods as a token of appreciation for participating.

HOW MANY PEOPLE WILL TAKE PART IN THIS STUDY?
We are inviting all physicians and scientists within the Department of Oncology and the Arnie Charbonneau Cancer Institute at the University of Calgary to participate.

WHAT WILL HAPPEN DURING THIS STUDY?

You are invited to participate in an anonymous survey that will explore the current diversity of the Department of Oncology and the Arnie Charbonneau Cancer Institute at the University of Calgary. This survey is powered by Qualtrics and will take place online via the survey link provided. We estimate that it will take approximately 15 minutes to finish the survey. Your data will be kept on file for 5 years. We aim to disseminate the results of this research 6 months after the survey is initially sent out.

WHAT WILL HAPPEN IF I CHOOSE TO WITHDRAW FROM THE STUDY EARLY?
Your participation is completely voluntary and no personally identifiable information such as your name, specific age or training program is collected. All data is stored on encrypted servers at the University of Calgary.

Because your responses are not linked to your email address, we will be unable to retract your answers should you choose to withdraw at a later time. Therefore, please do not submit this survey should you choose not to participate in this study.

No data that could be used to identify you as an individual will be published. All data where there is a small number of respondents that could be identifying of an individual participant will instead be published in aggregate with other measures to ensure confidentiality. Thorough consultation was undertaken by the designers of this survey with equity, diversity and inclusion researchers to ensure that the sensitive nature of the questions asked, and the data recorded will not be linked or identifiable to any one individual or small group to maintain anonymity. We therefore ask you to answer as honestly and openly as you are comfortable. All data is stored on encrypted servers and no individual answers will be shared with any institution.

Even though the likelihood that someone may identify you from the study data is very small, it can never be completely eliminated. Every effort will be made to keep your information be kept confidential, and to follow the ethical and legal rules about collecting, using and disclosing this information.

After the study is done, we will still need to securely store your data that was collected as part of the study. We will keep your data and study records stored for 5 years after the end of the study.

WILL I BE COMPENSATED FOR PARTICIPATING IN THIS STUDY?
As a token of appreciation for participating, you have the option of providing your email address (through a separate website that is in no way linked to your survey responses) for a chance to win Apple AirPods as a token of appreciation for participating.

WHAT ARE MY RIGHTS AS A PARTICIPANT IN THIS STUDY?
You have the right to be informed of the results of this study once the entire study is complete. If you would like to be informed of these results, please contact the researcher. Your rights to privacy are legally protected by federal and provincial laws that require safeguards to ensure

that your privacy is respected.

IS THERE ANY CONFLICT OF INTEREST RELATED TO THIS STUDY?
There are no conflicts of interests that the researchers have to report.

WHO DO I CONTACT FOR QUESTIONS RELATED TO THIS STUDY?
If you have questions about taking part in this study you should talk to the researchers or co-investigators.

These person(s) are:
Doreen Ezeife 403-521-3912
Amanda Khan   403-521-3642

If you have questions about your rights as a participant or about ethical issues related to this study and you would like to talk to someone who is not involved in the conduct of the study, please contact the Office of the Health Research Ethics Board of Alberta.   Telephone: 780-423-5727 Toll Free: 1-877-423-5727

Q2 **DO YOU AGREE TO PARTICIPATE IN THIS STUDY?**
BY PRESSING "YES" YOU CONSENT TO PROVIDING YOUR ANSWERS TO THE RESEARCH TEAM.

○ No (terminate study) (1)

○ Yes (continue on to study questions) (2)

End of Block: Default Question Block

Start of Block: Role and Demographics

Q3 ***Role and Demographics Questions***
Why are we asking? These questions allow us to better understand whom comprises the University of Calgary's Department of Oncology and Arnie Charbonneau Cancer Institute to provide context for the collected survey data.

Q4 **What is your current academic rank?**

- ○ Lecturer (1)
- ○ Assistant Professor or Clinical Assistant Professor (2)
- ○ Associate Professor or Clinical Associate Professor (3)
- ○ Full professor or Clinical Professor (4)
- ○ No academic appointment (5)

Q5 **What FTE best describes your employment/position? i.e. 0.5, 1.0**

____

Q6 **How long have you been a physician/scientist?**

- ○ Early career ((1)
- ○ Mid-career (5-15 years) (4)
- ○ Senior-career (>15 years) (5)

Q7 **Do you currently hold a leadership position? If so, please select where you hold your leadership position (select all that applies):**

- ▢ National or international level (1)
- ▢ University level (2)
- ▢ Institute level (3)
- ▢ Departmental level (4)

Q8 **What age bracket do you fall within?**

- ◯ 25-34 (1)
- ◯ 35-44 (2)
- ◯ 45-54 (13)
- ◯ 55-64 (14)
- ◯ 65-74 (15)
- ◯ 75+ (16)

Q9 **What is your marital status?**
Why are we asking? This question allows us to better understand how marital status affects experiences and opportunities within the field of oncology, and how these change over time in response to any future initiatives aimed at mitigating potential biases and inequities against, and/or providing supports for individuals with a given relationship status.

- ◯ Single (1)
- ◯ Married / in a domestic relationship (2)
- ◯ Divorced/separated (5)
- ◯ Widowed (6)
- ◯ I prefer not to answer this question (7)

Q10 **Do you have any children (under the age of 18) or dependents you are responsible for or share care for? If so, how many?**
Why are we asking? These questions allow us to better understand how providing care/support for family members – including family members defined as having an impairment – may impact

experiences and opportunities within the field of oncology, and how these change over time in response to any future initiatives aimed to support caregivers.

	Not Applicable
	0 1 2 3 4 5 5 6 7 8 9
Children ()	
Adults ()	

Q11 **Do any of your parents or guardians have a college/university degree?**

- ○ One (1)
- ○ Both (2)
- ○ Neither (3)
- ○ I prefer not to disclose this information (4)

Q12 **Please indicate your approximate household income when you were a teenager**

- ○ Less than $25,000 (1)
- ○ $25,000 to $50,000 (2)
- ○ $50,000 to $75,000 (3)
- ○ $75,000 to $100,000 (4)
- ○ $100,000 to $125,000 (5)
- ○ $125,000 to $150,000 (6)
- ○ $150,000 + (7)
- ○ I prefer not to answer (8)
- ○ I don't know (9)

Q13 **What degrees have you earned? Please select all that apply.**

- ☐ Undergraduate degree (5)
- ☐ MD or equivalent (eg: MBBS) (1)
- ☐ Masters or equivalent (3)
- ☐ PhD or equivalent (4)
- ☐ MBA or equivalent (6)

End of Block: Role and Demographics

Start of Block: Gender Identity and Sexual Orientation

Q14 **Gender Identity and Sexual Orientation**
Why are we asking? These questions allow us to better understand how gender identity and

sexual orientation affect experiences and opportunities within the field of oncology, and how these change over time in response to any future initiatives.

Q15 **Which of the following best describes your current gender identity/identities? (Select all that apply)**

[Agender] – Agender is a person who does not identify themselves as having a particular gender.

[Gender-fluid] - Gender-fluid is a nonbinary gender identity that's not fixed and is capable of changing over time. [Gender identity] – Gender identity means a person's internal sense of whether they are male, female, both or neither. It is a person's internal, deeply held sense of one's gender, and is not visible to others. Individuals may have more than one gender identity, and it can be fluid over time.

[Non-binary] - Nonbinary gender is an umbrella term to describe any gender identity that does not fit into the gender binary of male and female. Nonbinary gender (also sometimes referred to as genderqueer) people may, for example, identify as having no gender, fall on a gender spectrum somewhere between male and female, or identify as totally outside binary gender identities.

[Transgender] - Transgender refers to individuals whose gender identity or expression differs from societal expectations of the sex they were assigned at birth

[Two-spirit] - Two-spirit is an umbrella term used to describe an Indigenous person who does

not identify with colonial gender structures. It may be used to describe gender, sexual, and spiritual identity.

- ◯ Agender (1)
- ◯ Gender fluid (2)
- ◯ Man (3)
- ◯ Non-binary (4)
- ◯ Transgender (5)
- ◯ Two-spirit (6)
- ◯ Woman (7)
- ◯ I self-identify as… (please type response) (8) ____
- ◯ I do not know (9)
- ◯ I prefer not to answer (10)

Q16 **Which of the following best describes your sexual orientation(s)? (Select all that apply)**
[Asexual] - a spectrum that represents individuals who feel little to no sexual attraction
[Bisexual] - an individual attracted to more than one sex, gender, or gender identity
[Gay] - an individual who is emotionally, sexually, and/or romantically attracted to members of the same gender [Lesbian] - a woman who is physically, sexually, and/or emotionally attracted to another woman or female-gendered person
[Queer] – an umbrella term for the whole non-heterosexual community; queerness intentionally has no single definition beyond "not straight"
[Straight] - an individual who is emotionally, sexually, and/or romantically attracted to members of the opposite gender [Two-spirit] – an umbrella term used to describe an Indigenous person who does not identify with colonial gender structures. It may be used to describe gender, sexual, and spiritual identity

[Pan-sexual] – sexual, romantic, or emotional attraction towards people regardless of their sex or gender identity

- ▢ Asexual (1)
- ▢ Bisexual (2)
- ▢ Gay (3)
- ▢ Lesbian (4)
- ▢ Queer (5)
- ▢ Straight / Heterosexual (6)
- ▢ Two-spirit (7)
- ▢ I do not know / Questioning (8)
- ▢ I prefer not to answer (9)
- ▢ I self identify as.... (please type response) (10) ____
- ▢ Pansexual (11)

End of Block: Gender Identity and Sexual Orientation

Start of Block: Diverse Abilities in the Workplace and Learning Environment

Q17 **Diverse Abilities in the Workplace and Learning Environment**

Q18 **Do you view yourself as having a disability where the Accessible Canada Act defines disability as:**
"any impairment, including a physical, mental, intellectual, cognitive, learning, communication or sensory impairment - or a functional limitation - whether permanent, temporary or episodic in

nature, or evident or not, that, in interaction with a barrier, hinders a person's full and equal participation in society."

- ◯ Yes (1)
- ◯ No (2)
- ◯ I prefer not to answer this question (3)

Q19 **If so, what do you regard as your disability? (Select all that apply)**

- ▢ Not applicable (9)
- ▢ Blind/visual impairment (1)
- ▢ Deaf/hearing impairment (2)
- ▢ Speech/communication disability (3)
- ▢ Mental health disability (4)
- ▢ Mobility/physical disability (5)
- ▢ Emotional disability (6)
- ▢ Cognitive disability (7)
- ▢ A health condition that affects your ability to be a physician (8)
- ▢ I prefer not to answer this question (10)
- ▢ I prefer to self-describe, please specify: (11) ____

End of Block: Diverse Abilities in the Workplace and Learning Environment

---

Start of Block: Race, Ethnic and Cultural Origins and Religious Beliefs

Q20 **Race, Ethnic and Cultural Origins/Belongings and Religious Beliefs**
Why are we asking? These questions allow us to better understand how being racialized in a society, and how ethnicity and cultural origin affects experiences and opportunities within the field of oncology, and how these change over time in response to any future initiatives, particularly those around anti-discrimination.

Q21 **From your name, physical appearance and/or anything you always wear, are you easily identifiable as a visible minority?**

- ○ Definitely yes (1)
- ○ Probably yes (2)
- ○ Probably no (3)
- ○ Definitely no (4)
- ○ Not sure (5)
- ○ I prefer not to answer this question (6)

Q22 **The 2016 census defined visible minorities as being a member of one of the following groups. Please select as many categories as you identify with.**

- ▢ Arab (6)
- ▢ Black (3)
- ▢ Caucasian (17)
- ▢ First Nations (11)
- ▢ Chinese (2)
- ▢ Filipino (4)
- ▢ Inuk (Inuit) (12)
- ▢ Japanese (10)
- ▢ Korean (9)
- ▢ Latin American (5)
- ▢ Metis (13)
- ▢ South Asian (eg: Indian, Bangladeshi, Sri Lankan) (1)
- ▢ Southeast Asian (eg: Cambodian, Indonesian, Thai) (7)
- ▢ West Asian (eg: Iranian) (8)
- ▢ I prefer not to answer this question (14)
- ▢ Other (please specify): (15) ____

Q23 **What would you consider to be your religious or spiritual affiliation that you currently practice or associate with? Please select all that applies.**

- ▢ Atheist / Agnostic / No religious or spiritual affiliation currently practiced (1)
- ▢ Bahá'í Faith (2)
- ▢ Buddhism (3)
- ▢ Christianity (any, including Catholic, Protestant, Evangelical, etc.) (4)
- ▢ Confucianism (5)
- ▢ Hinduism (6)
- ▢ Jainism (7)
- ▢ Judaism (8)
- ▢ Islam (9)
- ▢ Native Spirituality (10)
- ▢ Sikhism (11)
- ▢ Spiritual (12)
- ▢ I prefer not to answer this question (13)
- ▢ Other (please specify): (14) ____

Q24 **From your name, physical appearance and/or anything you always wear, are you easily identifiable as a member of a specific religion?**

- ○ Definitely yes (1)
- ○ Probably yes (2)
- ○ Probably no (3)
- ○ Definitely no (4)
- ○ Not sure (5)
- ○ I prefer not to answer this question (6)

Q25 **What religion would people assume you belong to? Select all that applies.**

- ▢ Buddhism (1)
- ▢ Christianity (Catholic, Protestant, Anglican, Evangelical, etc.) (2)
- ▢ Confucianism (3)
- ▢ Hinduism (4)
- ▢ Judaism (5)
- ▢ Islam (6)
- ▢ Native Spirituality (7)
- ▢ Sikhism (8)
- ▢ Other (please specify): (9) ____

End of Block: Race, Ethnic and Cultural Origins and Religious Beliefs

Start of Block: Job Perceptions

Q26 **Job Perceptions**

Q27 **Thinking about the past year, how would you rate the culture of respect in your department?**
"Culture of respect" refers to the attitudes, behaviors, and standards of your colleagues as related to access to, inclusion of, and level of respect for individual and group needs, abilities, and potential across the spectrum of diverse backgrounds and identities.

- ◯ Excellent (1)
- ◯ Very good (2)
- ◯ Good (3)
- ◯ Adequate (4)
- ◯ Poor (5)
- ◯ Very Poor (6)

End of Block: Job Perceptions

Start of Block: Mentorship and Academics

Q28 **Mentorship and Academics**

Q29 **If you currently act as a formal or informal mentor to a trainee(s) and/or colleague(s), please specify how many trainees/colleagues you are currently mentoring. If you do not act as a mentor in this capacity, please select "Not applicable."**

Not Applicable

0 2 4 6 8 10 12 14 16 18 20

The amount of trainee(s) I mentor is: ()	
The amount of colleague(s) I mentor is: ()	

Q30 **Over the course of your career, how many trainees do you estimate that you've mentored? If you do not act as a mentor in this capacity, please select "Not applicable".**

	Not Applicable
	0 2 4 6 8 10 12 14 16 18 20
The amount of trainee(s) I mentor is: ()	

Q31 **How many peer-reviewed publications have you been an author on?**

- (1)
- 5-10 (2)
- 10-25 (3)
- 25-50 (4)
- 50-100 (5)
- >100 (6)

Q32 **Where have you presented your research before? Please select all that applies:**

- ☐ Local conference / research day (1)
- ☐ Provincial meeting / provincial society (2)
- ☐ National meeting / conference (3)
- ☐ International conference (4)
- ☐ I have not presented my research before (5)

Q33 **In dollar amounts how much grant funding have you received in the past 5 years?**

- ○ < $5,000 (1)
- ○ $5,000 - $10,000 (2)
- ○ $10,001 - $50,000 (3)
- ○ $50,001 - $100,000 (4)
- ○ >$100,000 (5)

Q34 **In dollar amounts how much grant funding have you received throughout your career?**

- ○ < $5,000 (1)
- ○ $5,000 - $10,000 (2)
- ○ $10,001 - $50,000 (3)
- ○ $50,001 - $100,000 (4)
- ○ >$100,000 (5)

End of Block: Mentorship and Academics

Start of Block: Personal Experience with Discrimination / Harassment

Q35 **Personal Experience with Discrimination**

Q36 **During the past five years, have you ever felt discriminated against because of any of the following while working at the University of Calgary?**
Discrimination is defined as unjust or prejudicial treatment based on the grounds of race, age, gender, sex, and/or other traits or characteristics. Discrimination includes but it is not limited to examples such as inequity in work assignments, evaluations/assessments, distribution of

resources/support, compensation, and hiring practices. Discrimination can be direct or indirect, subtle or overt. Please check all that apply.

- ☐ Gender (4)
- ☐ Age (5)
- ☐ Race/ethnicity (6)
- ☐ Sexual orientation (7)
- ☐ National origin (8)
- ☐ Disability (9)
- ☐ Religion (10)
- ☐ Marital status (11)
- ☐ Socioeconomic status (12)
- ☐ Pregnancy, childcare responsibilities, other caretaking responsibilities (13)
- ☐ Level of education (Masters vs Doctoral or other advanced degree) (14)
- ☐ Political view (15)
- ☐ Not listed (Please specify): (16) ____
- ☐ Not applicable (17)
- ☐ I would rather not disclose this information (18)

Q37 **How often did you feel that you experienced the above discrimination in the past 5 years while working at the University of Calgary?**

- Never (4)
- Once (5)
- 2 – 4 times (6)
- 5 – 10 times (7)
- Regularly / On an ongoing basis (8)

Q38 **Was the person who harassed / discriminated against you someone in a position to directly affect your academic, and/or professional opportunities?**

- Yes (4)
- No (5)
- Not sure (6)
- Does not apply (7)
- I would rather not disclose this information (8)

Q39 **Please rate your level of agreement with the following statement:**
"I understand how to and feel comfortable reporting harassment incidents at my workplace."

- Strongly disagree (4)
- Somewhat agree (5)
- Strongly agree (6)
- Does not apply (7)

End of Block: Personal Experience with Discrimination / Harassment

---

Start of Block: How Can We Improve?

Q40 **How Can We Improve?**

Q41 **What should the Department of Oncology / the Arnie Charbonneau Cancer Institute do to address mistreatment or harassment?**

____

Q42 **What should the Department of Oncology / the Arnie Charbonneau Cancer Institute do to advance equity diversity and inclusion in the workplace?**

____

Q43 **What should the Department of Oncology / the Arnie Charbonneau Cancer Institute do to make faculty hiring practices more equitable?**

____

End of Block: How Can We Improve?

Start of Block: SUBMIT

Q44 **Please press the forward button below if you are finished the survey and consent to your answers being used in aggregate in this research study. If you do not wish to submit your answers, please close your browser window now.**

End of Block: SUBMIT
